# Inhalable bacteriophage endolysins: a novel therapeutic strategy for drug-resistant bacterial pulmonary infections – a comprehensive review

**DOI:** 10.1080/07853890.2026.2681295

**Published:** 2026-06-26

**Authors:** Dongyan Ding, Hailing Duan, Fang Zhang, Xi Wang, Yijia Guo, Ruiping Tian, Xiaotian Dai, Haidong Li

**Affiliations:** a Department of Respiratory and Critical Care Medicine, Jiangbei Campus of The First Affiliated Hospital of Army Medical University(No. 958 Hospital of PLA Army), Chongqing, China; b Department of Respiratory Medicine, Southwest Hospital, Third Military Medical University (Army Medical University), Chongqing, China

**Keywords:** Bacteriophage endolysins, drug-resistant bacterial pulmonary infections, antimicrobial agent, drug-resistant bacteria

## Abstract

**Background:**

Pulmonary infections caused by multidrug-resistant (MDR) bacteria pose a severe global health threat with high mortality rates, especially in hospital-acquired pneumonia. The stagnation of new antibiotic development underscores the urgent need for alternative therapeutics.

**Methods:**

This review summarizes recent advances in the application of bacteriophage endolysins against major MDR respiratory pathogens, including *Staphylococcus aureus*, *Streptococcus pneumoniae*, *Pseudomonas aeruginosa*, *Acinetobacter baumannii* and *Klebsiella pneumoniae*. We focus on their mechanisms of action, synergistic effects with antibiotics, and efficacy against biofilms.

**Results:**

Endolysins demonstrate potent and species-specific lytic activity against a broad spectrum of MDR bacteria. A key advantage is their low propensity for inducing resistance. Critically, when administered *via* optimized inhalation delivery systems, endolysins can achieve high local concentrations in the lungs – though this depends on factors such as the aerosol device, formulation properties and patient-related variables – effectively eradicating pathogens in animal models of pneumonia with minimal systemic toxicity. This direct pulmonary delivery approach bypasses many challenges associated with systemic administration and enhances therapeutic outcomes.

**Conclusion:**

Endolysins represent a promising paradigm shift in combating drug-resistant bacterial pulmonary infections. Their rapid lytic activity, synergy with conventional antibiotics, and suitability for inhalable formulation position them as a potent adjunct or alternative therapy. While challenges in stabilization and large-scale production remain, advancing inhalation delivery systems for endolysins holds immense potential to holds immense potential to revolutionize the treatment of recalcitrant respiratory infections.

## 1. Introduction

Pneumonia and other types of respiratory infections caused by multidrug-resistant (MDR) or extensively drug-resistant (XDR) bacteria are among the most common and deadly infections globally. The burden of antimicrobial resistance (AMR) is particularly acute in the respiratory tract. According to the most recent Global Burden of Disease Study 2023, lower respiratory infections (LRIs) were responsible for 2.50 million deaths globally in 2023, with children under five and adults over 70 years carrying the highest burden [1]. Notably, *Streptococcus pneumoniae* accounted for 634,000 deaths (25.3% of all LRI deaths), followed by *Staphylococcus aureus* (271,000 deaths, 10.9%) and *Klebsiella pneumoniae* (228,000 deaths, 9.1%) [1].

The challenge of treating these infections is compounded by rising AMR. The World Health Organization’s 2025 Global Antimicrobial Resistance and Use Surveillance (GLASS) report reveals that approximately one in six bacterial infections worldwide are now resistant to antibiotics, with resistance rates highest in the WHO South-East Asian and Eastern Mediterranean Regions [2]. Critically, common Gram-negative pathogens responsible for pneumonia – including *K. pneumoniae, Acinetobacter* spp. and *Pseudomonas aeruginosa* – are becoming increasingly difficult to treat, even with last-resort antibiotics such as carbapenems [2,3]. This crisis stems from a convergence of factors, including the overuse and misuse of antibiotics, the remarkable genetic adaptability of bacteria, and a critical stagnation in the development of novel antibacterial drugs [4]. The mortality rate from resistant respiratory infections continues to rise, underscoring the dire and urgent need for alternative therapeutic paradigms beyond traditional antibiotics. With the advent of a potential ‘post-antibiotic era’, the threat of untreatable bacterial pneumonia looms large, prompting global health policy initiatives such as the updated WHO Global Action Plan on AMR (February 2026) to prioritize the development of innovative antimicrobial strategies [5].

Endolysins are promising antimicrobial agents against infections in humans and animals and biological control agents in food [6]. The term endolysins were first coined in the 1960s in a study of endolysins in *Staphylococcus aureus* [7]. As enzymatic products of bacteriophage genomes, endolysins hydrolyze peptidoglycan bonds in the bacterial cell wall, facilitating bacteriophage progeny release during the lytic cycle. This process of destroying the host bacteria is supported by the observation that adding endolysins to the sensitive bacteria without a bacteriophage caused the loss of over 90% of the target bacteria [8]. As a recombinant and purified enzyme, endolysins can rapidly cleave peptidoglycans, often in a species- or subspecies-specific manner, rendering it a promising antibacterial agent with a broader range of effects than bacteriophages. For example, bacteriophages targeting *S. aureus* are usually inactive against coagulase-negative *Staphylococcus*; in contrast, endolysins purified from these bacteriophages are active against the coagulase-negative bacteria [9]. Endolysins have several important features, such as novel ways of acting [10], high specificity [11], low risk of drug resistance [12], synergistic activities with different antibacterial agents, and the ability to function effectively on biofilms [13] and mucosal surfaces. Endolysins can kill susceptible organisms when applied as a recombinant protein exogenously, which is especially significant given the emergence of bacterial resistance to classical antibiotics [11]. Endolysins are more suitable than bacteriophages to be used as novel antimicrobial agents because they do not require lysogen and transduction, unlike bacteriophages [11]. Therefore, endolysins are promising alternatives for antibiotics [14], thus providing hope for the fight against antibiotic-resistant bacteria in the respiratory system (Table 1).

**Table 1.** Comparison of the advantages of chemical antibiotics, bacteriophage and endolysins in treating bacterial infections.

Comparison	Chemical Antibiotics	Bacteriophages	Endolysins
Substance	Chemical Molecules	Biological Entities	Biological Macromolecules
Specificity	Low host-specificity (Targets multiple classes e. g. Gram-negative and Gram-positive bacteria)	High host-specificity (strain specific)	Low host-specificity (Targets multiple classes e. g. Gram-negative and Gram-positive bacteria)
Security	Ab-related side effects	Safe, little side effects	Safe, little side effects
Antibiogram	Antibiogram dependent	Antibiogram independent	Antibiogram independent
Spectrum	Broad-spectrum	Narrow-spectrum	Broad-spectrum
Treatment	Empiric treatment	No empiric treatment	No empiric treatment
Efficacy data	Qualitative RCT’s	Little efficacy data	Little efficacy data
Pharmacology	Well-studied pharmacology	Less predictable pharmacology	Less predictable pharmacology
Antibiofilm properties	No	Yes	Yes
Resistance	Occurs	Occurs	Not observed
Discovery Process	Difficult	Easy	Moderate
Production costs	Low	Moderate	High
Development costs	High	Low	Moderate
Approval process	Well established	Regulatory Guidelines not (fully) established	Established (‘biologicals’)
Pharmacokinetics	Release controllable	Concentration increases due to replication	Rapid Clearance

Ab: antibody; RCT’s: randomized controlled trials.

While previous reviews have extensively covered the general antibacterial potential of endolysins, the novelty of this work lies in its focused discussion on the inhalable delivery of endolysins for treating drug-resistant pulmonary infections. This review uniquely synthesizes the latest preclinical and clinical advancements in this specific route of administration, critically analyses the associated formulation and delivery challenges – such as stability during nebulization and pulmonary immunogenicity – and integrates the emerging evidence on endolysins’ efficacy against secondary bacterial infections in post-COVID-19 patients. By providing a comprehensive and up-to-date perspective on the translational pathway of inhalable endolysin therapy, this review aims to bridge the gap between basic research and clinical application, offering valuable insights for future development in this promising field.

While inhalation delivery is rationally promising for achieving high local concentrations, it is crucial to note that the clinical translation of inhalable endolysins remains in its early stages, with all current evidence derived from preclinical models.

### 1.1. Literature search and selection methodology

Initial Search Period: A comprehensive literature search was conducted from database inception through 31 March 2024, using PubMed, Scopus and Web of Science. The following key search terms were used in various combinations: ‘endolysin’, ‘bacteriophage lysin’, ‘pulmonary infection’, ‘pneumonia’, ‘inhalation’, ‘nebulization’, ‘biofilm’ and the names of target pathogens (e.g. *Staphylococcus aureus*, P*. aeruginosa*, *S. pneumoniae*, *Acinetobacter baumannii, K. pneumoniae*).

Inclusion/Exclusion Criteria: We focused on primary research articles and authoritative reviews published in English, prioritizing studies on endolysin activity against the specified respiratory pathogens, with particular emphasis on inhalation delivery and biofilm disruption. The selection process aimed to capture foundational studies as well as recent, high-impact research published up to the cutoff date.

Post-Search Updates: During the manuscript revision process (2025–2026), we incorporated key studies published after the initial search cutoff that were directly relevant to the scope of this review, including the GBD 2023 Lower Respiratory Infections study (December 2025), the WHO GLASS Report 2025, and the latest preclinical combination therapy study by Wang et al. This ensures that the review reflects the most current evidence while maintaining methodological transparency regarding the original search timeline.

## 2. What are endolysins?

Peptidoglycan is the main structural component of the bacterial cell wall. Bacteriophage endolysins, also known as endolysins or murein hydrolases, are well-studied peptidoglycan hydrolases. These polysaccharide monomers comprise the disaccharide N-acetylglucosamine (NAG) and are linked to N-acetylmuramic disaccharide (NAM) residues *via* a short peptide bridge. Endolysins are highly evolved enzymes encoded by double-stranded DNA bacteriophages at the end of the cleavage cycle by attacking glycosidic or peptide bond activity to degrade peptidoglycan and subsequently release bacteriophage progeny [8,15].

Endolysins are similar to bacterial autolysins and exolysins in their structures and functions [16] and are closely related to a small family of mammalian peptidoglycan recognition proteins. Bacteria possess an auto-polysaccharide hydrolase that allows their cells to grow and divide [17]. Bacterial exolysins are produced and secreted by bacteria to kill the cells of different strains or species; in contrast, endolysins kill the species that produce them. Endolysins accumulate in the host cell independently of the bacteriophage virion and the associated holin protein. As endolysins have no independent signal sequences, they are regulated by different bacteriophage endolysins and holins during cell lysis [18]. They may effectively control pathogenic host bacteria without disturbing the normal microbial community [19].

### 2.1. Structure of endolysins

**Figure 1.**
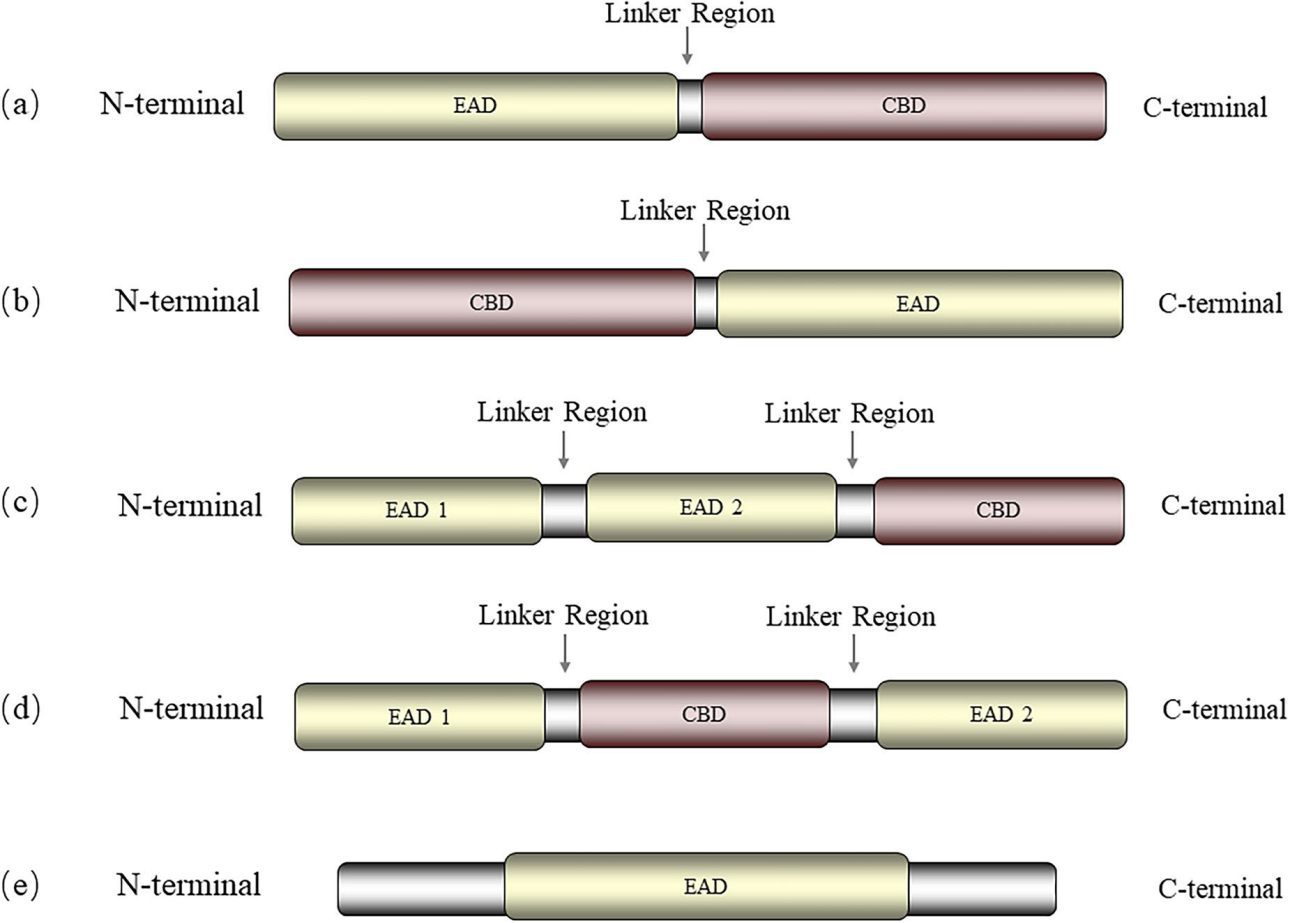
Modular models of common bacteriophage endolysins. Most were constructed from domains separated by the short junction Linker Region. (A) A model with an N-terminal enzyme active domain (EAD) and a C-terminal cell-wall-binding domain (CBD). (B) Modularized endolysins with C-terminal EAD and N-terminal CBD. (C) A multi-domain model with two EADs and one C-terminal CBD. (D) Multi-domain mode, with CBD located between two EADs. (E) A simple spherical model of EAD without CBD. Figure Generation Software: This figure was created using Science Slides.

Most endolysins (usually with a molecular weight of 15–40 kDa) are monomeric proteins with two domains (Figure 1). The N-terminal enzyme active domain cleaves various specific peptidoglycan bonds in the murein layer of host bacteria; in contrast, the C-terminal cell-wall binding domain (CBD) recognizes and binds different epitopes in the cell wall to properly anchor the catalysis of the enzyme active domain [20]. The catalytic domain is responsible for cleavage-specific covalent bonds in the peptidoglycan structure, which is essential for maintaining its intrinsic structural integrity. CBD imparts specificity to endolysins by non-covalent binding to species- or strain-specific epitopes associated with the cell envelope. The modular structure of endolysins is an important feature of its development as an antimicrobial agent. This can be achieved by exchanging endolysins domains to produce chimeras that alter binding specificity, enzyme activity, or both [21]. Endolysins of gram-positive bacteria usually consist of one or more enzyme domains and cell-wall-binding domains, whereas gram-negative lytic proteins usually comprise a single catalytic domain. Thus, the host range of gram-positive targets for endolysins tends to be narrower, whereas the range of targets for gram-negative endolysins is wider [22]. Bacteriophage-encoded cell-wall hydrolases include lysozyme (n-acetyl-paramidase), glycosidase (n-acetyl-β-d-glucosaminase), n-acetyl-paramidase acyl-l-alanine amidase and L-alanine-d-glutamic endopeptidase [23]. Endolysins usually consist of one of these four N-termini and are usually grouped according to their cleaved cell-wall peptidoglycan sites, with variations in the cell-wall-binding domain.

The high specificity resulting from the combined action of catalysis and CBD domains makes endolysins highly refractory to the resistance typically observed with classical antibiotic therapy [24]. Even if resistance develops, the use of these enzymes can be prolonged by domain shuffling or in combination with other endolysins or antibiotics. Amidase endolysins are derived from bacteriophages and endolysins of *Pneumococcus*, which contain a binding domain that is present in the cell wall of *Pneumococcus* [25]. Furthermore, when the chimeras were made of amidase catalytic domains, inactive pneumococcal cell walls, and known choline-binding domains, the new proteins were fully active in these cell walls [16].

### 2.2. Functional mechanism of endolysins

**Figure 2.**
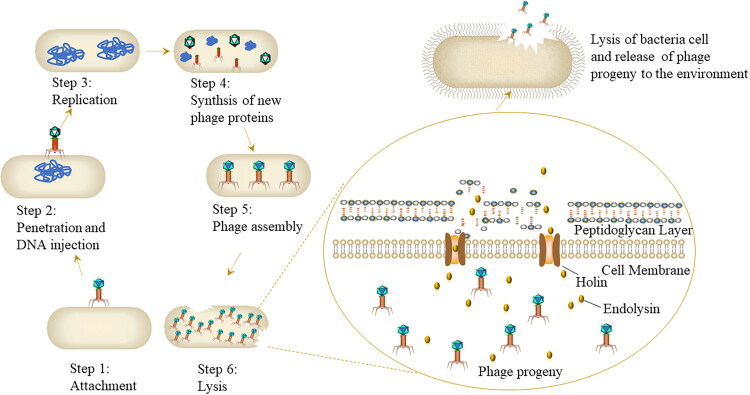
After specific recognition and attachment of the bacteriophage to the host cell (attachment), the viral genome is translocated into the cytoplasm of the bacteria (genomic translocation). The host copies genomic information and synthesizes and assembles bacteriophage proteins that form viral structures (bacteriophage replication). Later in the life cycle, specific bacteriophage endolysins are synthesized. The enzymatic activity of endolysins leads to the disintegration of the host envelope and eventually to host cell lysis and release of bacteriophage progeny (cleavage and release). The new bacteriophage can reattach and infect a new host cell, continuing the life cycle. Figure Generation Software: This figure was created using Science Slides.

The final phenomenon of bacteriophage infection is the release of progeny bacteriophages, usually carried out by two proteins: a murein-degrading enzyme called endolysins, which is essential for hydrolyzing peptidoglycan, and a tunnel-like protein called holin, which produces nonspecific pores in the plasma membrane of the cell to facilitate endolysins transport [26]. The bacteriophage lyses the host cell internally and is controlled by the holin − lysin system [27]. Holins are small proteins that accumulate in the membrane, and once they reach critical concentrations, they create holes in the plasma membrane of the cell through oligomerization [28]. Endolysins are expressed and accumulated in the cytoplasm of host cells [27], and the entry of their peptidoglycan substrates through entry pores leads to the immediate hypoosmotic dissolution of the bacteria and, ultimately, cell death [29]. When applied externally (as a purified recombinant protein) to bacterial cell walls, endolysins also degrade the peptidoglycan of susceptible organisms, causing ‘extrinsic cleavage’ [30]. Endolysins are essential for successful bacteriophage infection. The ‘cleavage from the outside’ caused by purified endolysins should not be confused with the cleavage of bacteriophage structural proteins, usually tail-related components of the virion, which locally degrade peptidoglycan after the bacteriophage particle has attached to the cell to inject the bacteriophage DNA into the host [31] (Figure 2).

## 3. Treatment basis of endolysins in MDR bacterial pulmonary infections

As treating lung infections by antibiotic-resistant bacteria becomes more difficult, new antimicrobial treatments are needed, either as standalone agents or in addition to antibiotics. Broad-spectrum antibiotics exert selective pressure on the target pathogens and symbiotic organisms. An important advantage of endolysins is their high specificity for certain peptidoglycan types [32]. Usually, endolysins only kill their host bacterial species or subspecies. For example, endolysins from Group C *Streptococcus* bacteriophage (PlyC) kill Group A, Group C and Group E *Streptococcus*. Nevertheless, they have no effect on the *Streptococcus* species and other gram-positive bacteria normally found in the human mouth [19]. Similar results were observed for *Pneumococcal*-specific endolysins; however, in this case, the enzyme was also tested against penicillin-resistant *Pneumococcal* strains with the same killing efficiency [33]. Lysozymes can only kill diseased organisms and have little effect on normal human bacterial flora. The targeting of endolysins allows single pathogens to be picked out from the bacterial queue and avoids unnecessary symbiotic death. The size of gram-positive endolysins is 25–40 kDa. In contrast, endolysins targeting gram-negative bacteria are in the range of 15–20 kDa and have a single catalytic structure. The structure of endolysins varies between enzymes targeting gram-positive and gram-negative bacteria, reflecting differences in cell wall structures between these major bacterial groups.

A significant challenge in treating chronic pulmonary infections, such as those in cystic fibrosis or ventilator-associated pneumonia, is the formation of bacterial biofilms. Biofilms are complex, structured communities of bacteria that adhere to biological or abiotic surfaces and are embedded within a self-produced extracellular polymeric substance (EPS) matrix composed of polysaccharides, proteins and DNA [34]. This mode of growth represents the predominant bacterial lifestyle in nature and is a key factor in the pathogenesis of chronic respiratory infections [35]. The EPS matrix acts as a physical barrier, protecting resident bacteria from host immune defenses and antibiotic penetration. Furthermore, bacteria within biofilms enter a metabolically dormant state, rendering them tolerant to conventional antibiotics that typically target actively dividing cells. This combined physical and physiological protection can result in biofilm-associated bacteria being up to 1,000-fold more resistant to antibiotics than their planktonic (free-swimming) counterparts [36]. Despite various therapeutic approaches, including high-dose combination antibiotic therapy and aggressive debridement where possible, the complete eradication of mature biofilms remains clinically elusive, often leading to persistent, recurrent infections and poor patient outcomes.

The peptidoglycan hydrolase activity of endolysins offers a distinct advantage here. By directly targeting the fundamental structural component of the bacterial cell wall, endolysins can disrupt the integrity of biofilms from within, lysing embedded bacteria in a way that many conventional drugs cannot. Unlike many antibiotics that require active bacterial metabolism to exert their effects, endolysins can kill metabolically dormant persister cells within biofilms, addressing a key mechanism of biofilm-associated tolerance.

The right combination of enzymes and antibiotics can help control antibiotic-resistant bacteria and resume using certain antibiotics that have conferred resistance on bacteria. In addition, endolysins can sometimes be used as a supplement to antibiotics. Synergies between these two types of antimicrobials can significantly reduce the dose required for some applications and improve therapeutic effects while reducing the risk of developing resistance by simultaneously attacking two different targets. Antibiotics’ cleavage of bacterial cell wall peptidoglycan and the increased sensitivity to endolysins may be the basis of endolysins-antibiotic synergy [37]. While there have been many successful treatment examples of endolysins alone as an antimicrobial agent, more than one endolysins have been used, or one endolysins has been used in combination with another class of antimicrobials to gain a synergistic effect against infection. The differences among the endolysins, antibiotics and bacteriophages are shown in Table 1.

### 3.1. Targets of gram-positive bacteria

Endolysins are most effective against gram-positive bacteria because they are particularly active in natural structures that lack protective outer membranes. Endolysins have been developed to fight many gram-positive pathogens, including *S. aureus* [38], *S. pneumoniae* [33], *Streptococcus pyogenes* [19] and *Bacillus anthracis* [39]. The efficacy of these endolysins has been demonstrated using *in vivo* models.

### 3.2. Staphylococcus aureus

*Staphylococcus aureus* is an opportunistic pathogen that inhabits human skin and mucous membranes and is a common cause of lung infections. Hospital-acquired methicillin-resistant *Staphylococcus aureus* (HA-MRSA) is an important cause of life-threatening nosocomial infections, such as ventilator-acquired pneumonia [40]. MRSA is a major antimicrobial-resistant variant/phenotype of *Staphylococcus aureus* resistant to the entire class of beta-lactam antibiotics. The emergence of MRSA and the rapid development of drug resistance make the clinical treatment of *Staphylococcus aureus* infections challenging [41]. Vancomycin-resistant MRSA has also been reported, undermining this last line of defense against MRSA infections [42]. Many bacteriophage endolysins and chimeric proteins are effective against *S. aureus* [43], although most have not yet been used to treat respiratory infections. However, they have been used for nasal decolonization, which can be helpful in preventing *Staphylococcus aureus* infections, especially those that occur after surgery [44].

The successful purification of MV-L clonal lysozyme encoded by the bacteriophage φMR11 of *S. aureus* led to the rapid and complete lysis of cells of tested *S. aureus* strains, including subpopulations of MRSA, vancomycin-resistant *S. aureus* and vancomycin-intermediate *S. aureus* (VISA). Synergies were observed when the endolysin MV-L was combined with glycopeptide antibiotics, such as vancomycin or teicoplanin, to eliminate MRSA artificially inoculated into the nostrils of mice in as little as 15 min [37]. In addition, one out of nine mice showed complete elimination of infection after treatment with MV-L [37].

ClyS is a chimera formed by fusing the N-terminal endopeptidase domain of bacteriophage Twort’s lysozyme with the C-terminal CWT domain of bacteriophage phiNM3 lysate protein, and it is effective against methicillin-resistant, vancomycin intermediate-resistant and sensitive strains of *Staphylococcus aureus* [45]. In a mouse nasal mucosal MRSA colonization model, ClyS reduced the potential for nasal colonization, with a greater than 2 log decrease observed 1 h after a single treatment compared to the treatment with buffer alone. Synergistic interactions between vancomycin and benzoxicillin were investigated to prevent death from systemic MRSA infections.

In a study on the efficacy of LysGH15, an endolysin derived from the staphylococcal phage GH15, in treating MRSA-infected pneumonia in mice, LysGH15 was administered intranasally 1 h after infection: the treatment group showed a survival benefit, and lung tissue obtained 24 h after infection showed lesser inflammatory cell infiltration than the control group tissue [46].

SAL200 is used to treat pneumonia and other respiratory infections caused by several different pathogens, such as *S. pneumoniae*, *Staphylococcus aureus*, *B. anthracis*, *A. baumannii* and P*. aeruginosa* [47]. SAL-1, a therapeutic agent for *Staphylococcus aureus* infections, was administered intravenously to prolong the viability of mice infected with MRSA. The results strongly support SAL200 as a highly effective anti-MRSA bactericide [48]. A recent study evaluated the efficacy of intranasal administration of SAL200 in treating MRSA-induced pneumonia in a mouse model of fatal pneumonia. The survival rate 2 h after infection was 90–95% in the treatment group and 10–40% in the control group [49]. Specific endolysins have been identified and purified to kill gram-positive bacteria after exposure [19,33]. While *Staphylococcus aureus* endolysins have not yet been used to treat pneumonia in humans, several clinical trials treated other staphylococcal infections with endolysins [43].

### 3.3. S. pneumoniae

*S. pneumoniae* remains the most common bacteria causing community-acquired pneumonia, but MDR bacteria challenge the management of pneumonia [50]. Colonization of the respiratory tract by *S. pneumoniae* is a prerequisite for establishing an infectious process. Successful colonization is critical for the horizontal transmission of antibiotic resistance and/or virulence-related genes [51]. Microbial colonization of the lower respiratory tract is associated with chronic obstructive pulmonary disease [52]. Eliminating this colonization would reduce the incidence of this disease. Several endolysins have been identified in the pneumococcal system, including palamidase [53], Cpl-1, Cpl-7, Cpl-7S and Cpl-711 lysozymes [54,55]. A purified pneumococcal bacteriophage lytic enzyme (Pal) could kill 15 common pneumococcal serotypes, including penicillin-resistant strains, within seconds of exposure [33]. This was confirmed in a nasopharyngeal-carrying mouse model [19]. Cpl-7S is an engineered variant derived from Cpl-7, in which 15 amino acid residues of CWBD are altered to enhance bactericidal activity [54]. Cpl-711 is a synthetic chimera that contains CD from Cpl-7 in the N-terminal and C-terminal regions [54]. Cpl-1 and Cpl-711 showed specific anti-pneumococcal activity against plankton and biofilm cultures; in contrast, Cpl-7S was effective against a wider range of susceptible bacteria and could kill other associated pathogens, including *S. pyogenes* and *Enterococcus faecalis* [54]. The most potent endolysins against *S. pneumoniae* is a chimeric protein: chimeric Cpl-711 significantly improved wild-type Cpl-1; engineered Cpl-7S was a highly effective endolysins against pneumococcal bacteria and provided a promising therapeutic prospect for the treatment of MDR pneumococcal infections [56].

Synergies can be observed when pneumococcal endolysins Cpl-1 is combined with certain antibiotics. In a mouse model of severe pneumococcal pneumonia, the inflammatory response was lower in mice treated with Cpl-1 and amoxicillin than in the untreated mice [57]. *In vitro* experiments also confirmed that Cpl-1 and gentamicin were increasingly synergistic with decreasing penicillin minimum inhibitory concentration in killing *pneumococcus*, whereas Cpl-1 and penicillin showed synergistic effects against penicillin-resistant strains [58]. Recent evidence further solidifies the potential of Cpl-1 as an ‘antibiotic resensitizing agent’. In a murine model of invasive penicillin-resistant pneumococcal disease, penicillin alone was ineffective. However, co-administration with Cpl-1 significantly improved survival rates to 89%, offering a novel combinatorial strategy for treating resistant pneumonia [59].

### 3.4. S. pyogenes

*S. pyogenes* (Group A beta-hemolytic *Streptococcus*) is a major human-specific bacteria that colonizes the pharynx, anal and genital mucosa. It is the main cause of bacterial pharyngitis [60]. Eradicating carriage reduces *Streptococcus*-associated disease, and an extensive antibiotic regimen that may increase streptococcal resistance has been the only treatment [61]. *S. pyogenes* was not detected 2 h after the oral administration of endolysins in heavily colonized mice. This method can eliminate or reduce streptococcal carriers or infections in the upper respiratory mucosal epithelium and associated diseases [19].

### 3.5. B. anthracis

*B. anthracis* is a small aerobic or facultative anaerobic gram-positive or gram-variable, coated, spore-forming rod. Inhalation of anthrax leads to the accumulation of *B. anthracis* spores in the alveoli. The spores are engulfed by immune cells and transported to regional lymph nodes, where the bacteria germinate, multiply and produce toxins that induce cell damage and death [62]. *Bacillus anthrax* can kill up to 99% of untreated victims. PlyG lysozyme isolated from the γ bacteriophage of *B. anthracis* specifically kills *B. anthracis* isolates and other members of its ‘cluster’ *in vitro* and *in vivo*. PlyG can be used for identifying and treating *B. anthracis* [39].

### 3.6. Targets of gram-negative bacteria

The protective outer membrane of gram-negative bacteria poses a significant barrier, preventing most natural bacteriophage endolysins from accessing their peptidoglycan target. This inherent obstacle makes developing endolysins against gram-negative pathogens more challenging than against gram-positive ones.

This outer membrane consists of phospholipids and lipopolysaccharides. Lipopolysaccharide molecules are held together by phosphoric acid bonds between the acidic sugars. The outer membranes of gram-negative bacteria prevent most endolysins from reaching peptidoglycans, making these endolysins less effective therapeutic agents [63]. However, some endolysins from bacteriophages of gram-negative bacteria can penetrate the bacterial outer membrane with the help of an amphiphilic tail found at the end of the molecule [64].

### 3.7. P. aeruginosa

*P. aeruginosa*, a major airway pathogen particularly in healthcare settings and in patients with cystic fibrosis, exhibits formidable antibiotic resistance, making it a prime candidate for novel therapies like endolysins. MDR infections by *P. aeruginosa* are associated with chronic recurrent respiratory infections (cystic fibrosis, chronic obstructive pulmonary disease, pneumonia and bronchiectasis). *P. aeruginosa* is the second most isolated organism in patients with ventilator-associated pneumonia [65]; the infection has a mortality rate of up to 30% [66]. *P. aeruginosa* can produce large amounts of alginate EPS (d-mannuronic acid-L-Guluronate), and its biofilm is destabilized by alginate-specific. *P. aeruginosa* can be more than 100 times antibiotic-resistant when grown in biofilm [67]. Endolysins have spiral peptides at the C-terminus, which may bind to the lipopolysaccharide of *P. aeruginosa* PAO1. Therefore, endolysins penetrate the bacterial outer membrane and degrade the peptidoglycan layer responsible for maintaining the cell structure. Subsequently, the N-terminus of endolysins, which may contain catalytic domains, approaches the peptidoglycan layer, leading to bacterial cell lysis. A complete conformation of endolysins may be necessary for effective function [68].

Endolysins, such as KZ144 and EL188, target and kill *P. aeruginosa* only when co-applied with a membrane permeator [69]. Owing to the difficulty of co-administration, some studies have targeted the engineering design of the enzyme itself [70]. Constructed by fusing endolysin KZ144 with the cationic antimicrobial peptide MAP-29, Art-175 represents a class of engineered ‘Artilysins^®^’. These chimeric proteins are designed not only to penetrate the protective outer membrane of gram-negative bacteria but also to enhance penetration through the negatively charged EPS of biofilms. This dual action leads to effective degradation of the peptidoglycan layer and killing of embedded, persistent cells within biofilms, which are often tolerant to conventional antibiotics [71].

Lysozymes are also capable of cracking G bacteria. These enzymes contain AMP-like elements (peptides with amphiphilic secondary structures and positive net charges) that destabilize the outer membrane. *Bacillus* amyloliquefaciens *Lys1521* is one of the first endolysins with natural cationic peptides to lyse *P. aeruginosa* cells [72]. Bacteriophage-derived LysPA26 was isolated without the use of an outer membrane permeator and showed lytic activity against MDR strains of *P. aeruginosa*. Its antibacterial activity was tested against other MDR bacteria, including *Enterococcus faecium*, *S. aureus*, *K. pneumoniae*, *A. baumannii* and *Escherichia coli* [73]. In addition, a mouse infected with *A. baumannii* was used to test LysSAP26’s *in vivo* antibacterial activity. The lysozyme reduced the bacterial population by up to four logarithmic units in 30 min and was demonstrated to be pH and thermally stable. In addition, OBPgp279 has anti-G-*P. aeruginosa* activity [74].

PlyPa91 is suitable for use in infected mucosal environments and protects against death in mouse models of pulmonary *P. aeruginosa* infection [64]. The delivery route significantly affected mouse survival in this model: 70% of mice treated with intranasal and endotracheal infusion combinations were protected. Endolysins PlyPa91 protected 70% of animals in models of *P. aeruginosa* pneumonia when administered intranasally and endotracheally, while survival dropped to 20% when the enzyme was administered intranasally alone. However, *in vivo* evidence for eliminating *P. aeruginosa* from the respiratory tract after endolysins treatment is still insufficient.

### 3.8. A. baumannii

*A. baumannii* is a critical-priority pathogen notorious for causing difficult-to-treat hospital-acquired infections, particularly in intensive care units [75]. It is a potential respiratory pathogen (especially in immunocompromised patients). This opportunistic pathogen can cause community and nosocomial infections, especially in critically ill patients in intensive care units. It is a major cause of ventilator-associated pneumonia and can cause severe nosocomial infections [76]. Its remarkable resistance to antibiotics, including carbapenems [77], is caused by its ability to form biofilms, which may explain its survival properties, increased virulence and MDR [78]. The incidence of coexisting lower respiratory tract infections with *A. baumannii* strains secondary to SARSCoV-2 infection accounted for approximately 1% of COVID-19 hospital admissions worldwide during the pandemic [79,80]. Recent studies in 2023 highlight that secondary bacterial infection in COVID-19 patients, particularly those caused by carbapenem-resistant *A. baumannii* and MRSA, have risen to 15-20% in ICU settings, further exacerbating the urgency for alternative therapies like endolysins [81]. Therefore, new antimicrobials are needed to overcome resistant bacteria and biofilms.

Several studies have described the broad-spectrum endolysins of *A. baumannii* and found evidence that they can kill more parental bacteriophage-specific strains [82]. One study showed that the novel endolysins LysSS exhibits broad antibacterial activity against a variety of MDR bacterial species, including *Salmonella*, carbapenem-resistant *A. baumannii*, *P. aeruginosa, K. pneumoniae and S. aureus*; thus, LysSS endolysins, which can target gram-positive and gram-negative bacteria, may have therapeutic potential [82]. Endolysins of *A. baumannii* PD-6A3 lysed 70.5% (141/200) of clinical MDR *A. baumanni* isolates; this endolysins has also shown lysing activity against *Enterococcus*, methicillin-resistant *S. aureus, K. pneumoniae, P. aeruginosa* and *E. coli* [83]. LysAB2 is a broad-spectrum enzyme that is active against gram-positive and gram-negative bacteria, including *A. baumannii, E. coli and S. aureus* [84]. Compared with other endolysins, Art-Top3 acts very quickly in serum, has high stability, exerts high bacterial activity against clinical MDR *A. baumannii* strains *in vitro* and *in vivo*, and is less toxic to primary human cells *in vitro* [85].

An *in vitro* and *in vivo* study combining colistin with endolysins ElyA1 for treating infections caused by MDR pathogens showed higher activity in minimal inhibitory concentrations (MICs) and time-kill assays in various strains of *A. baumannii* and *P. aeruginosa* but not *K. pneumoniae* in a mouse model of lung infection [86]. LysABP-01 endolysins combined with colistin increased growth inhibition and synergistic effects against drug-resistant strains of *A. baumannii*; combinations with other antibiotics showed no such growth inhibition [87]. However, evidence from animal experiments and human lung infection clinical trials is still insufficient and needs further improvement.

### 3.9. K. pneumoniae

*K. pneumoniae* colonizes the respiratory tract and can cause infection when the human immune system is weakened, or the flora is unbalanced in the body, leading to a variety of diseases with high morbidity and mortality [88]. The emergence of extended-spectrum beta-lactamases, coupled with carbapenem-resistant bacteria, can render the treatment and control of *K. pneumoniae* infections significantly more challenging [89]. LysG24 and LysCA were shown to be effective in a mouse model of pneumonia infected with *K. pneumoniae*, in which LysG24 and LysCA were both effective in reducing lung inflammation. LysCA was more effective based on clinical symptoms and bacterial load in mouse lungs [90]. Ethylenediaminetetraacetic acid (EDTA) is required to invade and destroy host bacteria effectively.

Hence, endolysins have extensive lytic activity against gram-positive and gram-negative bacteria [14]. Related preclinical studies have confirmed their efficacy and safety in the respiratory tract and drug-resistant bacterial infections. If applied outside of bacterial cells as recombinant proteins, endolysins can induce rapid lysis and cell death without holins or other companion enzymes, which are desirable characteristics of antimicrobial agents. Endolysins are unlikely to replace chemical antibiotics in clinical practice, but they offer a potential solution in the fight against AMR. In some cases, antibiotics do not need to be completely replaced but can be applied with endolysins, making them more effective at lower doses and giving them a second life. Nonetheless, further human clinical trials of endolysins are required. The key preclinical *in vivo* studies demonstrating the efficacy of these endolysins against major respiratory pathogens are summarized in Table 2.

**Table 2.** Summary of key preclinical *in vivo* studies of endolysins for respiratory pathogens.

Endolysin	Target pathogen	Infection model	Route of administration	Key findings	Ref.
Cpl-1	*S. pneumoniae* (PSSP & PRSP)	Murine lethal pneumonia	Inhalation	Treatment starting 24h post-infection reduced mortality by 80%; significantly reduced bacterial load in lungs and rapid resolution of inflammation.	[99]
Cpl-1 + Penicillin	*S. pneumoniae* (PRSP)	Murine invasive pneumonia/bacteraemia	Intraperitoneal	Combination therapy improved mouse survival to 89%, compared to ineffective penicillin monotherapy, demonstrating ‘antibiotic resensitization’.	[122]
Cpl-711 (Chimeric)	*S. pneumoniae*	Murine bacteraemia	Intraperitoneal	Showed superior efficacy in reducing bacteraemia levels and improving survival compared to its parent endolysin Cpl-1.	[54,55]
SAL200 (contains SAL-1)	MRSA	Murine lethal pneumonia	Intranasal	Administration 2h post-infection resulted in 90-95% survival in treated groups vs. 10-40% in controls.	[49]
LysGH15	MRSA	Murine pneumonia model	Intranasal	Administration 1h post-infection provided a survival benefit and reduced inflammatory cell infiltration in lungs.	[46]
PlyPa91	*P. aeruginosa*	Murine pneumonia model	Intranasal + Endotracheal	Combined delivery protected 70% of animals, significantly higher than intranasal delivery alone (20%).	[63]
Art-175 (Engineered)	*P. aeruginosa* (MDR & persisters)	*Galleria mellonella* larvae model	Local injection	Showed highly efficient bactericidal activity *in vivo* against MDR strains and antibiotic-tolerant persisters.	[70]
Cpl-1 + Gentamicin or Cpl-1 + Pal	*S. pneumoniae*	Murine lung infection	Inhalation	Combination therapy with Cpl-1 and gentamicin or Pal showed enhanced efficacy compared to monotherapy; first demonstration of inhaled endolysin combination therapy; highlights importance of formulation for inhaled delivery.	[101]

## 4. Endolysins lung delivery

The activity of endolysins on the airway surface determines their lysis ability. The difference in the efficacy of endolysins therapy is related to its delivery method: orally, intravenously, or directly (topically) to a selected organ or site of infection. The endolysins movement pathway can be simple (topical application) or somewhat complex (systemic application of non-haematologically associated bacteria).

### 4.1. Oral administration

Oral administration is usually the most convenient and acceptable method for patients. Most oral drugs on the market come in tablet or liquid forms. However, for protein-based therapeutics such as endolysins, the oral route presents formidable barriers. Stomach acid contains proteases, including pepsin, trypsin, chymotrypsin and various peptidases, that can readily degrade endolysins; many endolysins contain cleavage sites that can be targeted by these enzymes [91]. If this acid and enzymatic barrier in the gastrointestinal tract is not overcome, delivery remains inefficient. The encapsulation of endolysins within protective carriers (e.g. liposomes, polymeric nanoparticles) has been proposed as a strategy to circumvent this problem [92].

Importantly, even if endolysins survive the gastrointestinal environment and are absorbed into the bloodstream – a significant challenge in itself – they would then face a second major barrier: proteolytic degradation in the systemic circulation. Blood contains numerous proteases (e.g. plasmin, thrombin and various serum peptidases) that can rapidly hydrolyze protein therapeutics, leading to short half-lives and reduced bioavailability. This dual-barrier problem – degradation in both the gastrointestinal tract and the bloodstream – fundamentally limits the feasibility of oral delivery for native, unmodified endolysins.

An *in vivo* study of endolysins was first reported in 2001, using a mouse model system to demonstrate that oral administration of endolysin C1 (derived from the bacteriophage C1 murein hydrolase) significantly reduced upper respiratory colonization of mice by *Streptococcus*, without affecting other microbes in the body [19]. However, this study focused on mucosal colonization rather than systemic infection, and the observed effects likely resulted from local activity in the oropharynx rather than absorption into the circulation.

In summary, oral administration of unmodified endolysins is not an effective strategy for delivering therapeutic concentrations to the systemic circulation or to deep lung tissues. Given that these protein drugs must pass through the stomach and digestive system – and, if absorbed, must then survive in the bloodstream – they are subjected to multiple levels of proteolytic degradation that compromise their integrity and bioavailability. Future development of orally administered endolysins will likely require advanced formulation strategies (e.g. PEGylation, fusion to stabilizing domains, or encapsulation in protease-resistant nanoparticles) designed to protect against degradation in both enteric and systemic compartments.

### 4.2. Absorption *via* injection

In the literature, endolysins have most often been intravenously administered to human subjects in clinical trials. Injection proved to be a highly effective way to deliver endolysins to the system, as it was the easiest way to overcome important body defense barriers. Commonly available injection routes include intraperitoneal, intramuscular and subcutaneous routes, or direct intravenous injections, which are used for respirator-associated infections, especially in preclinical trials.

A mouse model of pneumococcal transnasal infection was established and treated with endolysins Cpl-1 *via* a repeated intraperitoneal injection after infection [57]. According to various clinical measurements and lung morphological changes, the mice developed severe pneumonia at 24 h. When treatment was started at 24 h and every 12 h after that, 100% of the mice survived fatal pneumonia and showed rapid recovery. Cpl-1 significantly reduced the bacterial count in the lungs and prevented bacteraemia.

The first clinical trial using an endolysins-based drug was successful [93]. SAL200 is a recombinant form of endolysins SAL-1 that targets antibiotic-resistant *Staphylococcus* species. It has been reported that SAL200 is suitable for intravenous administration. A single dose of SAL200 was administered to 34 healthy male volunteers, and no serious side effects were reported throughout the study, indicating a good safety profile [93]. In addition, endolysins showed favourable pharmacokinetic characteristics. These findings suggest that endolysins therapy may be a safe and viable treatment option, although further research is recommended.

The injection delivers endolysins effectively and quickly into the bloodstream. The intravenous route presents fewer obstacles than other routes. However, intravenous administration is more invasive than the traditional oral route and requires patient visits to specialist clinics, which can be time-consuming and expensive. The intravenous site also carries potential complications, such as secondary bacterial infections at the injection site [94].

### 4.3. Local application

Local infection treatment can be used as an alternative to entering and exiting the bloodstream by applying the drug directly to the lungs rather than entering the bloodstream first. It can avoid the host’s immune system interactions and cause systemic toxicity. In contrast, topical application avoids the loss associated with absorption and distribution, increasing the potential of the lung to achieve endolysins density. Recent studies have demonstrated the feasibility of endolysin topical formulations, such as hydroxethylcellulose gels for wound infections [95]. Injecting bacteriophages directly into the site of bacterial infection also avoids adsorption and distribution problems but is more invasive than topical application. Local delivery to the lungs can be achieved through the endolysins inhalation treatment process, although this may also result in systemic delivery. The development of targeted delivery strategies, including lung-directed peptide-fused lysins, has further enhanced the potential of local endolysin therapy for pulmonary infections [96].

### 4.4. Atomizing inhalation

Atomization is widely used in respiratory medicine to deliver therapeutic drugs to the deepest airway surface by generating aerosols from liquid suspensions *via* vibrating mesh, compressed air (jet atomization), or ultrasound [97,98]. Nebulized inhalation enables direct drug deposition in the lower respiratory tract, achieving high local concentrations while minimizing systemic exposure – a critical advantage for treating deep-seated pulmonary infections and low exposure to systemic circulation, which can reduce systemic toxicity [99].

Inhalation of Cpl-1 has been investigated as a therapeutic approach for treating pneumococcal pulmonary infections. An early study by Doehn et al. demonstrated that inhaled Cpl-1 effectively reduced bacterial counts in the lungs and prevented bacteraemia in a mouse model of pneumococcal pneumonia [100]. Despite transient increases in inflammatory cytokines shortly after Cpl-1 inhalation, treated mice recovered rapidly, as evidenced by weight gain, and the elimination of inflammatory infiltration in the lungs led to an 80% reduction in mortality. This foundational study confirmed the safety of pulmonary endolysin delivery and showed no evidence of adverse effects [100]. More recently, Wang et al. advanced this work by investigating inhalable Cpl-1 formulations in combination with either gentamicin or the pneumococcal endolysin Pal in a murine lung infection model [101]. This study is particularly significant as it represents the first evaluation of combination inhaled endolysin therapy. The authors demonstrated that Cpl-1, when formulated for inhalation and administered together with gentamicin or Pal, exhibited enhanced antibacterial efficacy compared to monotherapy. These findings are clinically relevant for several reasons: (i) they support the use of endolysins as part of combination regimens to potentially reduce the risk of resistance emergence; (ii) they highlight the importance of formulation science in optimizing inhaled protein therapeutics; and (iii) they provide contemporary *in vivo* evidence that extends and reinforces the earlier proof-of-concept studies [101]. The synergistic activity observed between Cpl-1 and gentamicin is particularly noteworthy, as it suggests that inhaled endolysins could be used to potentiate the activity of conventional antibiotics against resistant pneumococcal strains. Endolysin Cpl-1 was developed as an inhalable powder to treat pneumococcal lung infections [102].

Wang et al. [103] conducted an innovative feasibility study on the antibacterial activities of Cpl-1 and ClyJ-3, administered by atomization, against *S. pneumoniae* lung infections. The evaluation was conducted using fluidic or vibrating mesh atomization, focusing on antibacterial activity, protein structural changes, and aerosol properties before and after atomization. The results showed that the biological activity of ClyJ-3 was completely lost owing to atomization. No significant difference was observed in the bactericidal activities of Cpl-1 before and after mesh atomization. However, spray atomization reduced the biological activity of Cpl-1, and the antibacterial activity was completely lost. In conclusion, inhalation delivery of endolysins is feasible; nonetheless, this depends on the protein and nebulizer, with the mesh nebulizer being preferred.

Topical use was at least three times more effective than systemic use. Inhaled drugs allow high doses of the drug to be delivered directly to the lungs; increasing the local drug density can increase treatment success without simultaneously increasing toxicity [104]. Therefore, direct pulmonary delivery is a reasonable and clinically relevant method for treating pulmonary infections by antibiotic-resistant bacteria. However, atomization should be modified to improve the stability of the agents and, thus, the viability of clinical trials of existing treatments. Nevertheless, atomized inhalation may result in coughing, decreased saturation, hypoxaemia, bronchoconstriction or bronchospasm, and occlusion of the expiratory filter may cause cardiac arrest [105]. Rigorous *in vivo* studies of its use are still lacking, and some fundamental questions remain to be answered.

It is important to contextualize these promising preclinical findings within the current clinical landscape. As of January 2026, no clinical trials investigating the inhalation of endolysins for treating pulmonary infections are registered on ClinicalTrials.gov. The foundational human safety data for endolysins, such as that from the completed Phase 1 trial of intravenous SAL200 (NCT03089697), support systemic administration but do not address the specific pharmacokinetics and local tolerability of pulmonary delivery. Therefore, inhalation is presently a compelling developmental, not yet clinical, route. The promising results from animal models underscore the urgency of initiating such clinical trials to bridge this translational gap.

## 5. Limitations of endolysins in treating pulmonary infections by antibiotic-resistant bacteria

While endolysins have many advantages in treating infections by antibiotic-resistant bacteria in the lungs, there are also associated problems that may not significantly hinder their use.

### 5.1. Delivery challenges

A major translational hurdle lies in optimizing endolysins delivery; enzymatic degradation by pulmonary proteases and rapid clearance by alveolar macrophages significantly reduce bioavailability Endolysins are easily degraded by the body’s natural defense mechanisms. Several viable endolysins have been isolated to fight lung pathogens; however, delivering them effectively to the site of infection is not a simple task. The lungs contain many immune cells and enzymes that can rapidly target proteins and peptides. Endolysins can be degraded by proteases or alveolar macrophages [106]. This can lead to poor bioavailability and irreversible damage to the structural integrity of the proteins.

### 5.2. Immunogenicity

Non-human proteins are effective inducers of specific immune responses, including immunoglobulin G (IgG) reactions and allergic reactions mediated by IgE. Endolysins are immunogenic proteins that, like other exogenous protein products, can initiate an immune response by activating T-cells and producing neutralizing antibodies [107]. Another limitation of endolysins is their short half-life *in vivo* due to the proteolysis of plasma proteases and degradation of enzyme aggregates [93]. Therefore, if endolysins are to be used systemically, they must be administered frequently *via* intravenous infusion, or modified to extend their half-life [108]. Immune responses may limit the effectiveness of non-human protein therapies. In particular, the development of neutralizing IgG antibodies after initial exposure to protein drugs *in vitro* may lead to subsequent treatment failure using the same therapy.

The impact of rabbit hyperimmune serum, targeting *Pneumococcal*-specific Cpl-1, on lysate activity was analyzed to confirm the effect of immune response on inactivation of endolysins [109]. The pharmacokinetics of Cpl-1 in the blood showed that the antibodies produced during Cpl-1 treatment were not neutralized without reducing the therapeutic effect, and the activity of Cpl-1 was independent of penicillin resistance. Cpl-1 did not visibly harm the mice when repeatedly administered at high doses. These results were also confirmed by using staphylococcal-specific endolysins [37].

A study was conducted to evaluate the immunogenicity of PlyC in human and mouse models [110]. PlyC induced effective IgG production in mice; immunogenic regions were present in PlyCA. In humans, approximately 10% of the population shows IgG responsiveness only to the PlyCB subunit. Despite its immunogenicity, PlyC induces a normal immune response without hypersensitivity, and no PlyC-specific IgE is detected [110].

MV-L effectively eliminated MRSA inoculated into the nostrils of mice; however, repeated administration of MV-L evoked a detectable level of humoral response in mice, and the antibodies did not eliminate the bacteriolytic activity, and the animals showed no adverse events after the administration of multiple doses of endolysins [37]. Antibodies against endolysins have high titres but non-neutralizing effects against MV-L and *Staphylococcus* ClyS endolysins [45]. Moreover, although repeated LysGH15 administration triggered the production of Lysgh15-specific antibodies in mice, these antibodies did not block lysate activity (nor LysGH15-binding ability) *in vitro* [111].

A study examining the safety of two endolysins, PaI and CpI-1, which target *S. pneumoniae*, showed that IgG levels increased in mice. Nonetheless, the immune cells had no adverse effects on the catalytic ability of endolysins [112].

As noted earlier, endolysins interact with the host immune system and cause the induction of antibodies, which is usually expected of protein-based drugs. However, antibodies cannot neutralize the catalytic activity of endolysins and do not significantly affect treatment efficacy. It is unclear why antibodies fail to neutralize the activity of endolysins. Still, endolysins are known to bind epitopes on bacterial surfaces with nanomolar to picomolar affinity, which may trump antibody binding [37,45]. Therefore, endolysins can be used repeatedly as therapeutic agents without degrading their activity, owing to antibody response.

Poor immune response to endolysins can reduce their effectiveness in the body or cause a systemic allergic reaction [113]. Studies using traditional mouse models have reported unreliable immunogenicity of human endolysins. Rats, dogs, monkeys and humans produced varying antibodies in preclinical and clinical trials of SAL200 [93]. Further studies are needed to understand the immunogenicity of endolysins.

### 5.3. Security

From a safety standpoint, the high specificity of endolysins is one of their most beneficial properties. An important advantage of endolysins over conventional antibiotics is their high specificity for certain peptidoglycans, which limit their antibacterial action to members of a certain bacterial genus, species, or even serotype [32]; symbiotic bacteria or accompanying microbial communities are not affected [30].

The potent lytic activity of endolysins raises the question of how to improve their purification and application to make them be used as therapeutic agents, especially against gram-positive bacteria, in which peptidoglycans are not covered by an outer membrane [6]. These endolysins show rapid killing kinetics and are active in high concentrations of salt and urea at a pH of 5.0 to 10.0 [64]. Endolysins are less active against gram-negative bacteria because of the presence of an outer membrane barrier [114]. However, given the reality and costs associated with obtaining regulatory approval for each new protein therapy, the narrow spectrum of cleavage activity exhibited by many of these enzymes poses a significant constraint on drug development [115]. For example, the host ranges of Pal and Cpl-1 are unique to *S. pneumoniae*. Therefore, the identification of highly active endolysins is required to overcome the financial barriers associated with developing these enzymes.

Endolysins have received increasing attention as an effective alternative to conventional antibiotics, but few studies have described the safety and toxicity of endolysins. SAL200 is a pharmaceutical compound containing the SAL-1 endolysins that targets *S. aureus*. In general toxicity studies, no adverse effects were observed or reported in rodents, dogs, or monkeys with single and repeated intravenous administration of endolysins SAL200. Pharmacological studies have shown no signs of toxicity in the central nervous system or respiratory function tests [116,117]. A human phase-1 study of SAL200 [93], the first human study of a drug based on bacteriophage endolysins, evaluated the pharmacokinetics, pharmacodynamics and tolerability of intravenous infusion of a single increasing dose of SAL200 in healthy male volunteers. No clinically significant values or trends were observed in clinical chemistry, haematology, coagulation analysis, urinalysis, electrocardiography (ECG), vital signs, or physical examination results. SAL200 was well-tolerated, and no serious adverse events were observed [93].

A safety study of pneumococcal endolysins demonstrated for the first time that Pal and Cpl-1 do not produce IgE reactions associated with hypersensitivity and anaphylaxis [112]. These enzymes do not activate specific immune pathways, such as apoptosis or inflammatory responses, thus strongly supporting the safety of Pal and Cpl-1.

Another study tested the effect of LysGH15 on *S. aureus* in mice and reported that high-dose LysGH15 injection did not cause significant adverse reactions or pathological changes in the major organs of the treated animals, nor did it raise total levels of the antibody IgE in the serum. A dedicated safety study on the pneumococcal endolysins Pal and Cpl-1 demonstrated that these enzymes did not induce IgE antibodies associated with hypersensitivity or anaphylaxis, nor did they activate the complement system, providing strong immunological support for their safety [111].

In the systemic administration of drugs to humans or animals, a major concern is the release of pro-inflammatory cell fragments associated with bacterial lysis, such as tetracyclic acid, lipoteichoic acid and peptidoglycan, which can lead to serious complications, including septic shock and multiple organ failure [118]. In a previous study, inflammation induced by LysGH15 administered simultaneously with MRSA was evaluated using an intraperitoneal approach. The mRNA levels of IFN-γ, IL-4 and IL-6 peaked at 12 h after MRSA injection. In addition, these levels were higher than those in MRSA-infected mice that had not been treated with endolysins. In the lysogen-treated group, cytokine mRNA levels subsequently decreased to normal at 24 h [119]. Peak increases in these cytokines 6–12 h after injection are consistent with a rapid decline in bacterial cells [100].

Preclinical trials using animal models have shown that endolysins has a good safety record without adverse reactions, such as fever, abdominal pain or diarrhoea [116]. Finally, owing to its protein properties, endolysins are non-corrosive and biodegradable [17].

### 5.4. Drug resistance

Broad-spectrum antibiotics accelerate the distribution of drug-resistant genes within bacterial communities by applying selective pressure to target pathogens and symbiotic organisms [17]. Antibiotics usually inhibit the basic metabolic pathways of bacteria, resulting in cell death. However, bacteria have adopted alternative ways to overcome antibacterial exposure [120]. Bacterial strains develop bacteriophage resistance through mutations, receptor modification, passive adaptation, restrictive modification, CRISPR-Cas and pseudo-lysogens [121]. The co-evolution of the bacteriophage and its host is thought to cause endolysins to bind to and cut into a highly conserved and immutable target in the cell wall, rendering it challenging for bacteria to evolve resistance to endolysins without compromising the integrity of the cell [108]; this could make the development of drug resistance a rare event, ensuring the survival of the bacteriophage [24]. There have been no reports of bacterial resistance to endolysins [86].

A study of *S. aureus* exposed to continuous diluents of endolysins LysGH15 showed no spontaneous resistance mutants: *S. aureus* developed no resistance after repeated exposure to LysGH15 [111]. Another study tested the effects of SAL200 endolysins on staphylococcal infections in humans and found that the likelihood of resistance to SAL200 was significantly lower than that to conventional antibiotics [93]. The report further showed that no mutants developed resistance to SAL200: *S. aureus* strains were repeatedly exposed to half the MIC of SAL200 30 times and failed to produce resistant mutants [93]. Pal-resistant *pneumococcus* cannot be detected after high Pal enzyme exposure [33]. One study evaluated endolysins KZ144 fused with the antimicrobial peptide MAP-29 and observed that continued exposure to engineered endolysins did not lead to the development of AMR in *P. aeruginosa* [71]. With a few exceptions, such as lysozyme or lysostaphin, pnagus proteins rarely develop resistance in their bacterial hosts, mainly owing to the prolonged horizontal gene transfer between pnagus and host systems [14].

### 5.5. Clinical application

As detailed in Table 3, the clinical translation of endolysins, particularly for pulmonary delivery, is at an early stage. A search of the U.S. National Institutes of Health clinical trials registry (ClinicalTrials.gov) using the term ‘endolysin’ reveals a limited number of registered interventional studies. As of January 2026, these include a completed Phase 1 safety study of the intravenous anti-staphylococcal endolysin candidate SAL200 (NCT03089697) [93], an ongoing/active study investigating a topical endolysin (NCT04191148) for decolonization of S. aureus, and a previously listed but now withdrawn/cancelled trial. Critically, no clinical trials investigating the inhalation of endolysins for treating pulmonary infections are currently registered. This starkly contrasts with the promising preclinical data and underscores that inhalation delivery remains a developmental, not yet clinical, route for endolysin therapy. Clinical trials are necessary to demonstrate that safety and efficacy data generated in preclinical laboratory settings can be reliably translated into clinical practice. Nevertheless, the difference between the amount of preclinical data and active recruitment in clinical trials for endolysins is significant. Compliance with Good Manufacturing Practice (GMP) is the gold standard for ensuring the quality, safety and efficacy of pharmaceutical products, whether investigational or approved, during manufacturing. When developed as a drug, endolysins must be stable, soluble and capable of hydrolyzing peptidoglycans, leading to challenges in generating endolysins products.

**Table 3.** Overview of endolysin clinical development.

Candidate/name	Target pathogen/ indication	Route of administration	Highest stage	Key findings / status	Note/clinical trial ID
SAL200 (contains SAL-1)	*S. aureus* (including MRSA) bacteraemia	Intravenous	Phase 1 Completed	Well-tolerated after single dose in healthy volunteers; no SAEs; PK profile supportive of further development.	NCT03089697 [92]
CF-301 (Exebacase)	*S. aureus* (including MRSA) bacteraemia	Intravenous (+ background antibiotic)	Phase 2/3	Phase 2 study completed, showing potential efficacy in combination with standard care. Phase 3 planned/ongoing.	Multiple trials registered
XZ.700 / MEndoC	Cutaneous T-cell Lymphoma (topical)	Topical	In Clinical Development	In preclinical/early clinical development for CTCL as a topical immunotherapeutic agent.	Non-infectious indication, demonstrating versatility
For Pulmonary Infections	Various MDR respiratory pathogens	Inhalation	Preclinical Stage	As of early 2026, no registered clinical studies. Substantial animal model data (see Table 2) provides proof-of-concept for initiating such trials.	A key future direction

The results of safety assessments and immunogenicity studies largely support the idea of endolysins as a potential therapy; however, the use of endolysins to treat infections by antibiotic-resistant bacteria in the lungs remains understudied, and further research in this area is needed to ensure that the different classes of endolysins do not pose a significant risk to the host.

### 6.6. Challenges in large-scale production and formulation

The translational pathway of endolysins faces significant industrial challenges. The development of cost-effective, high-yield expression systems (e.g. in E. coli) is paramount. Subsequent steps require optimizing protein solubility, achieving correct folding and ensuring stability during purification. Scaling up under GMP standards adds considerable complexity and cost. Finally, formulating endolysins into stable, biologically active powders or solutions suitable for nebulization presents a distinct set of formulation hurdles that must be overcome for successful inhalable product development.

## 6. Concluding remarks

The escalating crisis of MDR bacterial pulmonary infections demands a paradigm shift beyond traditional antibiotic development. This review has delineated the compelling case for bacteriophage endolysins, particularly *via* inhalable delivery, as a novel therapeutic strategy poised to address this urgent need. Their unique mechanism of action – direct enzymatic cleavage of peptidoglycan – confers rapid, bactericidal activity against a spectrum of resistant pathogens, including Staphylococcus aureus, *S. pneumoniae*, *P. aeruginosa* and *A. baumannii*, with a notably low propensity for inducing resistance. Critically, their efficacy extends to disrupting biofilms, a key barrier in treating chronic respiratory infections.

Unlike conventional antibiotics that often fail to penetrate the EPS matrix, endolysins hydrolyze the pep­tidoglycan layer of biofilm-embedded bacteria, effectively lysing both metabolically active and dormant persister cells within the biofilm community [15,43]. This direct cell wall-targeting mechanism bypasses the physical and physiological tolerance mechanisms that render biofilms refractory to standard antibiotic therapy.

The inhalation route emerges as the most rational and promising delivery strategy for pulmonary infections. As reviewed, direct pulmonary administration maximizes therapeutic concentration at the site of infection while minimizing systemic exposure and off-target effects on the commensal microbiome. Preclinical studies, such as the inhalation of Cpl-1 for pneumococcal pneumonia, have demonstrated significant survival benefits and reduced pathology, validating this approach *in vivo*.

Looking forward, the clinical translation of inhalable endolysins should be strategically focused. We posit their primary value not as a wholesale replacement for antibiotics, but as a potent adjunctive therapy for deep-seated, biofilm-associated and MDR infections where conventional treatments fail. A transformative perspective is their role as ‘antibiotic resensitizing agents’. Recent evidence, such as the synergy between Cpl-1 and beta-lactams against resistant *S. pneumoniae*, demonstrates that endolysins can restore the efficacy of existing antibiotics, potentially reclaiming drugs rendered obsolete by resistance. This synergy could revolutionize combination regimens, especially for ventilator-associated or post-viral (e.g. post-COVID-19) bacterial pneumonias where MDR pathogens are prevalent.

The path to the clinic, however, is punctuated by defined translational hurdles. These include optimizing protein stability and activity in inhalable formulations (powders or solutions), conducting rigorous early-phase clinical trials to establish safety and pharmacokinetics for pulmonary delivery, and scaling production under GMP. The promising preclinical efficacy and favourable safety profile summarized herein justify focused investment to overcome these barriers.

In conclusion, the convergence of endolysins’ unique bactericidal properties with targeted inhalation delivery represents a frontier in anti-infective therapy. By leveraging their precision, synergy with antibiotics, and biofilm-penetrating capability, engineered inhalable endolysins hold immense potential to become a critical weapon in the future arsenal against drug-resistant respiratory infections, ultimately improving outcomes for patients with otherwise untreatable pulmonary diseases. The future of this field lies in advancing these molecules from promising preclinical agents to essential clinical therapeutics.

## Data Availability

Data sharing is not applicable to this article as no data were created or analyzed in this study.
